# Hair loss in the era of CGRP inhibition: emerging evidence, mechanisms, and clinical implications

**DOI:** 10.3389/fneur.2026.1949826

**Published:** 2026-09-14

**Authors:** Adelė Antanaitytė, Mantas Jokubaitis, Jorinta Jokubaitė, Kristina Ryliškienė

**Affiliations:** 1 Faculty of Medicine, Vilnius University, Vilnius, Lithuania; 2 Centre of Dermatovenerology, Vilnius University Hospital Santaros Klinikos, Vilnius, Lithuania; 3 Faculty of Medicine, Vilnius University, Institute of Clinical Medicine, Clinic of Neurology and Neurosurgery, Vilnius, Lithuania

**Keywords:** calcitonin gene-related peptide, calcitonin gene-related peptide receptor antagonist, monoclonal antibodies, alopecia, drug-related adverse reactions

## Abstract

**Introduction:**

Calcitonin gene-related peptide (CGRP)-targeting therapies have transformed migraine management and are generally associated with a favorable safety profile. However, accumulating real-world evidence suggests a potential association with alopecia that was not systematically captured in pre-approval clinical trials. This review aimed to synthesize current evidence on alopecia associated with CGRP-targeting therapies, with a focus on clinical patterns, pharmacovigilance signals and underlying pathophysiological mechanisms.

**Methods:**

A structured literature search of PubMed was conducted to identify relevant evidence on CGRP-targeting therapies and hair loss through August, 2026. Eligible studies included clinical trials, observational studies, pharmacovigilance analyses and case reports reporting alopecia outcomes. Reference lists were screened for additional relevant studies. Due to heterogeneity of study designs and predominance of low-level evidence, findings were synthesized narratively without formal quality assessment.

**Results:**

Evidence from pharmacovigilance data and case-based observations suggests a reproducible signal of predominantly reversible, non-scarring alopecia occurring within weeks to months after initiation of CGRP-targeting therapies. Reports span multiple monoclonal antibodies and gepants, supporting a potential class effect. However, the available evidence is largely derived from spontaneous reporting systems and case series, limiting causal inference and precluding reliable estimation of incidence. Proposed mechanisms include neuroimmune dysregulation, impairment of hair follicle immune privilege, reduced microvascular perfusion, and disruption of the CGRP-insulin-like growth factor-1 axis.

**Conclusion:**

Alopecia has emerged as a potentially clinically relevant safety signal associated with CGRP-targeting therapies. Although the association is biologically plausible, the available evidence does not establish causality, frequency or the typical clinical course of this adverse event. Most cases appear to be mild, non-scarring and reversible. Given the expanding use of these therapies, increased clinical awareness and individualized management are essential. Future prospective studies with standardized dermatological assessment are needed to better define incidence, risk factors and underlying mechanisms.

## 1 Introduction

Calcitonin gene-related peptide (CGRP) plays a central role in the pathophysiology of migraine through its involvement in nociceptive transmission and vasodilation within the trigeminovascular system (1, 2). The development of CGRP-targeting therapies, including monoclonal antibodies and small-molecule receptor antagonists (gepants) has substantially improved the preventive and acute management of migraine and is generally associated with a favorable safety profile (3). However, increasing use of these agents in real-world clinical practice has led to the emergence of adverse events not systematically captured in pre-approval clinical trials. Pharmacovigilance analyses based on the Food and Drug Administration Adverse Event Reporting System (FAERS) and accumulating case reports have identified a potential signal of alopecia associated with CGRP-targeting therapies. Although the absolute incidence remains uncertain, the consistency of reports across different agents suggests that hair loss may represent a class-related safety signal rather than an isolated drug-specific effect (4–7).

The plausibility of this association is supported by the extra-neuronal functions of CGRP. Beyond its role in migraine, CGRP is widely expressed in the skin, where it contributes to neuroimmune regulation, maintenance of hair follicle immune privilege and microvascular homeostasis (8, 9). Disruption of these pathways may predispose susceptible individuals to hair follicle dysfunction and subsequent hair loss.

Despite growing interest, the relationship between CGRP inhibition and alopecia remains incompletely understood. Available evidence is largely derived from spontaneous reporting systems and small case series, which are inherently limited by reporting bias and lack of standardized dermatological assessment.

This review aims to provide an integrated overview of alopecia associated with CGRP-targeting therapies by combining pharmacovigilance signals, clinical evidence, mechanistic hypotheses and to translate these findings into practical considerations for clinical decision-making.

## 2 Materials and methods

This narrative review was conducted using a structured literature search in PubMed database up to 17 August 2026. The following Boolean search strategy was used: ((“calcitonin gene-related peptide”[tiab] OR CGRP[tiab] OR erenumab[tiab] OR fremanezumab[tiab] OR galcanezumab[tiab] OR eptinezumab[tiab] OR rimegepant[tiab] OR atogepant[tiab] OR ubrogepant[tiab] OR zavegepant[tiab]) AND (alopecia[tiab] OR “hair loss”[tiab] OR “telogen effluvium”[tiab] OR “alopecia areata”[tiab])). No additional PubMed filters were applied. The final search retrieved 31 records. Two reviewers independently screened titles and abstracts and assessed potentially eligible full-text publications. Disagreements were resolved by consensus.

Studies were considered eligible if they reported alopecia or hair loss in patients receiving CGRP-targeting therapies, including monoclonal antibodies and gepants. Clinical trials, observational studies, pharmacovigilance analyses and case reports or case series were considered. Review articles were used to identify and contextualize relevant literature, but were not considered primary evidence. Publications that did not report hair-loss outcomes in association with CGRP-targeting treatment, studies unrelated to CGRP-targeting therapies, and preclinical or pathophysiological studies without treatment-associated alopecia data were excluded from the core evidence synthesis. Eleven publications directly addressing alopecia or hair loss in association with CGRP-targeting therapies met these criteria and formed the principal evidence base of this review. Given the heterogeneity of the available evidence and the predominance of spontaneous pharmacovigilance reports, case reports, and observational data no formal risk-of-bias or methodological quality assessment was performed. The evidence was therefore synthesized narratively, with emphasis on the consistency of reported safety signals, clinical patterns, potential confounding factors, and biological plausibility (Table 1).

**Table 1:** Comparison of studies reporting alopecia as an adverse event associated with anti-CGRP medications.

Author, year	Sun et al. (4)	Ruiz et al. (5)	Woods (6)	Evers et al. (14)	Esguerra et al. (24)	Liang et al. (7)	Lee et al. (16)	Lanteri- Minet et al.(40)	Sessa et al.(28)	Argyriou et al. (41)	Jedlowski et al. (42)
Data source, period	FAERS, 2018 Q1–2023 Q1	2 clinical cases, FAERS (period N/A)	FAERS, period until 2021 Q4	Case series	Case report	VigiAccess, period until 2024-03-31, FAERS, 2020 Q1–2023 Q4	FAERS, period until 2023 Q2	One-year prospective real-world cohort study. FHU InovPain registry, France.	FAERS, 2018 Q1-2020 Q2	Prospective, multicenter, real-world Greek registry December 2020 – December 2022	FAERS, 1969–2021
Number of reports, gender distribution	*N* = 65,792. Male, *n* = 7,646 (11.62%), Female, *n* = 47,431 (72.09%), Unknown, *n* = 10,715 (16.29%), Alopecia reports, *n* = 1928	*N* = 2. Both female	*N* = 55,994. Alopecia reports *n* = 1827, Female *n* = 1,284 (70.68%), Male/unknown *n* = 543 (29.7%)	*N* = 279. All female. Alopecia reports, *n* = 6	*N* = 1, male	*N* = 23,542. Alopecia reports with rimegepant, *n* = 51, Alopecia reports with atogepant, *n* = 48	*N* = 81,145. 55,324 reports on mAbs 7,815 reports on gepants	*N* = 144 140 received ≥1 erenumab dose Female, *n* = 115 (82.1%), Male, *n* = 25 (17.9%). Alopecia reports, *n* = 1 (0.7%)	*N* = 23,312 Female, *n* = 15,099 (64.8%), Male, *n* = 2,657 (11.4%) Unknown, *n* = 5,556 (23.8%) Exact alopecia reports N/A, alopecia co-reported with constipation *N* = 144	*N* = 204 Female, *n* = 171 (83.8%) Male, *n* = 33 (16.2%) Alopecia reports, *n* = 2 (1.0%)	The total number of FAERS cases has not been reported. Percentage of female alopecia cases: erenumab 95.99%, fremanezumab 92.57%, galcanezumab 96.72%, eptinezumab 100%
Age	Mean 48.66 (14.96)	33 and 69	Mean 46	40–62	50	Mostly between 45 and 64	18–65	Mean 50.9 ± 11.4 years Range 19–75 years	Median 50 years	Mean 47.2 ± 10.9 years Range 22–70	N/A
Migraine type	Episodic and chronic	Episodic and chronic	Episodic and chronic	Chronic	Chronic	Episodic and chronic	Episodic and chronic	Mostly chronic migraine – 88.6%. Remaining cases consisted mainly of high-frequency episodic migraine.	Episodic and chronic	High-frequency episodic migraine (HFEM), n = 97 (47.5%); chronic migraine (CM), n = 107 (52.5%)	N/A
Acute migraine medication	N/A	Rimegepant	Ubrogepant	N/A	N/A	Ubrogepant, rimegepant	Ubrogepant, rimegepant	N/A	Disproportionality analyses were performed using triptans.	N/A	Rimegepant, Ubrogepant.
Preventative anti-CGRP medication	Erenumab, galcanezumab, fremanezumab, eptinezumab.	Erenumab, fremanezumab, galcanezumab, atogepant.	Erenumab, fremanezumab, galcanezumab, eptinezumab, rimegepant, atogepant.	Erenumab, galcanezumab, fremanezumab	Fremanezumab	Atogepant, rimegepant	Erenumab, galcanezumab, fremanezumab, eptinezumab, atogepant, rimegepant.	Erenumab.	Erenumab, fremanezumab, galcanezumab.	Fremanezumab	Erenumab, fremanezumab, galcanezumab, eptinezumab
Alopecia characteristics	N/A	Telogen effluvium	N/A	N/A	Scarring alopecia	N/A	N/A	N/A	N/A	N/A	N/A
Alopecia management	N/A	Intralesional triamcinolone injections, clobetasol solution.	N/A	Switching to another anti-CGRP mAb	Discontinuation of anti-CGRP mAb	N/A	N/A	N/A	N/A	N/A	N/A

FAERS, U. S. Food and Drug Administration Adverse Event Reporting System; mAb, monoclonal antibody; N/A, not available. Several pharmacovigilance studies included in this table used overlapping spontaneous reporting datasets, particularly FAERS. Therefore, report counts from these studies should not be interpreted as independent samples. Clinical case reports/case series are listed separately, but overlap with spontaneous reporting databases cannot be excluded.

## 3 Results and discussion

### 3.1 Clinical spectrum of alopecia

Alopecia is a heterogeneous dermatological condition broadly classified into non-scarring (reversible) and scarring (irreversible) forms (10). Although alopecia is not inherently a medically harmful condition, it has been linked to significant psychological consequences, including anxiety, depression, reduced self-esteem and negative body image, which can ultimately impair overall quality of life (6). The most prevalent forms are androgenic alopecia, telogen effluvium, alopecia areata and traction alopecia. Androgenic alopecia is a genetically predetermined and progressive form of hair loss affecting men and women. It is caused by a sensitivity to dihydrotestosterone which leads to shortened hair growth cycles and thinner, shorter hair (11). Telogen effluvium is characterized by rapid, diffuse hair shedding within a relatively short period. It is usually observed a few months after a significant physical or emotional stress and may also be triggered by sudden hormonal fluctuations or intake of drugs (12). Alopecia areata is an autoimmune disease in which the body’s immune system mistakenly attacks healthy hair follicles, stopping hair growth, which results in bald patches on the scalp (10). Traction alopecia is a type of progressive hair loss resulting from chronic, repeated pulling tension on hair follicles, often associated with tight hairstyles (13).

In the context of CGRP-targeting therapies, reported cases predominantly resemble diffuse, non-scarring hair shedding consistent with telogen effluvium, although in rare cases alopecia areata-like presentations have also been described (5, 6). This distinction is clinically relevant, as it may reflect differing underlying mechanisms, prognosis and management strategies.

### 3.2 Evidence of alopecia associated with CGRP-targeting therapies

Available evidence suggests a consistent pharmacovigilance signal linking CGRP-targeting therapies with alopecia. The majority of reports are characterized by diffuse, non-scarring hair shedding that typically develops within 2 weeks to 3 months after initiation of anti-CGRP therapy (5–7). Most reported cases have occurred in women, mainly as diffuse telogen effluvium or alopecia areata-like pattern. This effect appears to be reversible, with resolution typically occurring within 1–2 months following drug discontinuation (4–6, 14). However, the predominance of reported cases among women should not be interpreted as evidence of greater female susceptibility to CGRP inhibitor-associated alopecia. Migraine is substantially more prevalent in women, who consequently constitute a large proportion of patients receiving CGRP-targeting therapies (15). Significant signals of alopecia have been identified for the entire class of CGRP inhibitors, more frequently than in reports on other drugs (16). In one large dataset, alopecia accounted for approximately 3.26% of all reported adverse events associated with these therapies (6). This suggests that alopecia may be reported more frequently with anti-CGRP drugs than would be expected based on the overall FAERS background, although the absolute risk at the patient level remains unclear (6). A retrospective case series conducted by Evers et al. describes several cases of alopecia in patients treated with anti-CGRP monoclonal antibodies and emphasizes that hair loss may persist even after switching to a different drug of the same class, supporting a possible class effect rather than an issue with a single drug (14).

Across studies, alopecia has been reported in association with all major CGRP monoclonal antibodies, including erenumab, galcanezumab, fremanezumab and eptinezumab, as well as with gepants (4–6, 16). While erenumab accounts for the highest number of reported cases (almost 1,200 cases of alopecia), this likely reflects longer market availability and greater cumulative exposure rather than a true drug-specific risk (5, 6). In comparison, galcanezumab (554 cases of alopecia) and fremanezumab (175 cases of alopecia) are associated with fewer alopecia reports, whereas eptinezumab demonstrates the weakest signal of alopecia among the four anti-CGRP monoclonal antibodies, but a potential class effect remains likely (4–6). Signals have also been identified for gepants with some analyses suggesting a relatively higher reporting proportion for rimegepant compared with other agents, although differences in prescribing patterns, duration of exposure and reporting behavior likely contribute to these findings (7).

An important consideration when interpreting alopecia reported during CGRP-targeting therapy is the presence of potential confounding factors inherent to the migraine population. Patients receiving these therapies commonly have chronic migraine or severe episodic migraine and may experience substantial physiological and psychological stress, sleep disturbances, and exposure to multiple concomitant medications. Chronic stress is a recognized trigger of telogen effluvium and may also exacerbate alopecia areata. In addition, preventive medications frequently used in patients with migraine, including certain antidepressants, beta-blockers, and antiepileptic drugs, have been associated with hair loss (10). Consequently, the temporal occurrence of alopecia after initiating CGRP-targeting therapy does not by itself establish a causal drug–event relationship. Therefore, the potential contribution of underlying disease burden, stress, and concomitant pharmacotherapy should be considered when evaluating individual cases.

Differences in pharmacological targeting may theoretically contribute to variation in alopecia reporting across CGRP-targeting therapies. Erenumab differs from fremanezumab, galcanezumab and eptinezumab by targeting the CGRP receptor rather than the CGRP ligand, whereas gepants are small-molecule CGRP receptor antagonists with pharmacokinetic and tissue-distribution properties distinct from monoclonal antibodies (17). These differences may result in variability in the extent and duration of CGRP pathway inhibition and could therefore differentially affect physiological CGRP functions in peripheral tissues, potentially including the hair follicle. However, there is currently no direct evidence demonstrating that receptor versus ligand targeting determines alopecia risk and differences in pharmacovigilance reporting frequencies should not be interpreted as evidence of a pharmacological class effect or differential causal risk. However, these findings must be interpreted with caution. No prospective, controlled studies have systematically evaluated the incidence of alopecia in patients treated with CGRP-targeting therapies. The current evidence base is dominated by pharmacovigilance data and case reports, which are inherently subject to reporting bias, underreporting and lack of denominator data. Disproportionality signals in FAERS do not establish causality and may be influenced by notoriety bias, particularly as awareness of this potential adverse effect increases. Furthermore, alopecia is a highly prevalent condition with multifactorial etiology, making it challenging to distinguish drug-induced hair loss from coincidental occurrence.

### 3.3 Pathophysiological considerations

Several plausible mechanisms have been proposed to explain the potential association between CGRP inhibition and alopecia, integrating neuroimmune, neurovascular and growth factor–dependent pathways.

Experimental studies have shown that human scalp hair follicles express CGRP receptors and CGRP itself can protect hair follicles from interferon-γ–induced immune privilege collapse *in vitro*. These protective effects are likely diminished when CGRP signaling is inhibited (18, 19). The disruption of hair follicle immune privilege is considered to be the central event in the pathogenesis of alopecia areata (18, 20). CGRP in the skin affects not only blood vessels, but also keratinocytes, melanocytes, Langerhans cells, mast cells and other immune system cells modulating cytokine profile and immune status of the skin (8). A reduction in CGRP and related immunoregulatory signals favors T-helper 1 and T-helper 17 dominance. This may promote hair follicle immune privilege failure thereby increasing the risk of triggering autoimmune hair follicle damage or exacerbating a pre-existing predisposition to alopecia (18, 21).

In addition to immune modulation, CGRP is a potent vasodilator and contributes to the regulation of cutaneous microcirculation (16, 21). Experimental models of alopecia areata have demonstrated reduced CGRP levels accompanied by compromised blood supply to hair follicles, suggesting that hair loss may be triggered by relative vasoconstriction associated with decreased CGRP activity (4, 16, 22). Given the potent effect of CGRP as vasodilator, its pharmacological inhibition may reduce microvascular perfusion and thereby increase the susceptibility of hair follicles to ischemic and metabolic stress (6, 23). This mechanism is likely to represent a subtle, local follicular-level microcirculation disturbance significant to structurally and functionally susceptible “borderline” hair follicles. The clinical case described by Esguerra et al. supports this concept – locally high anti-CGRP concentrations at the injection site were associated with a pronounced inflammatory reaction in the absence of CGRP immunomodulatory effects and reduced microcirculation, leading to scarring alopecia through a combination of neuropeptide signaling blockade, local inflammation and vascular compromise (24).

Another proposed pathway involves the CGRP–insulin-like growth factor 1 (IGF-1) axis. IGF-1 is a key anagen-promoting growth factor secreted by dermal papilla cells. It plays a crucial role in regulating hair follicle cell proliferation, migration and the maintenance of follicular integrity through anti-apoptotic signaling (25). Experimental studies with mice have demonstrated that stimulation of sensory nerves increases CGRP expression, which in turn up-regulates IGF-1 expression in the skin and hair follicles. Importantly, these effects are absent in CGRP-deficient models, indicating that CGRP is required for IGF-1 induction in the cutaneous environment (22, 26). Given the well-established role of IGF-1 in maintaining anagen phase and dermal papilla function, pharmacological blockade of CGRP may reduce local IGF-1 availability and thereby contribute to impaired hair follicle support and premature transition to catagen and telogen phases (25, 27). Concomitant loss of CGRP-mediated vasodilation may further reduce microvascular blood flow to the follicle, amplifying the functional IGF-1 deficit (21, 25).

Collectively, these hypothetical mechanisms provide a biologically plausible explanation for anti-CGRP therapy-associated alopecia. Rather than representing independent processes, they are likely to interact and converge on common pathways affecting hair follicle homeostasis. Their integration may help explain the typically reversible nature of alopecia observed in clinical practice, suggesting a functional rather than permanent structural disruption of hair follicle activity (5, 8, 28).

### 3.4 Clinical implications and management considerations

Although the frequency and causal relationship of alopecia during CGRP-targeting therapy remain uncertain, hair loss may be clinically relevant because of its psychosocial impact and the expanding use of these agents. A structured, cause-oriented approach to management is essential (Figure 1). First, alternative causes of hair loss, such as nutritional deficiencies, endocrine disorders, systemic illness and concomitant medications should be carefully excluded (10, 12). This step is particularly important given the high baseline prevalence of alopecia in the general population. Telogen effluvium is estimated to affect up to 30% of adults at least once during their lifetime, while alopecia areata has a lifetime risk of approximately 2% worldwide (10). Second, if a drug-related etiology is suspected, individualized treatment decisions should be made based on the severity of hair loss, patient preference and migraine control. In mild cases consistent with telogen effluvium, continuation of anti-CGRP therapy may be appropriate with close monitoring and patient reassurance (12, 14). In more pronounced or persistent cases, dose adjustment, switching to an alternative CGRP-targeting agent or transitioning to a different preventive therapy may be considered, although evidence supporting these strategies remains limited (14). Third, referral to a dermatologist is recommended in cases of diagnostic uncertainty, atypical presentation or suspected alopecia areata, where targeted immunomodulatory treatment may be required.

**Figure 1:**
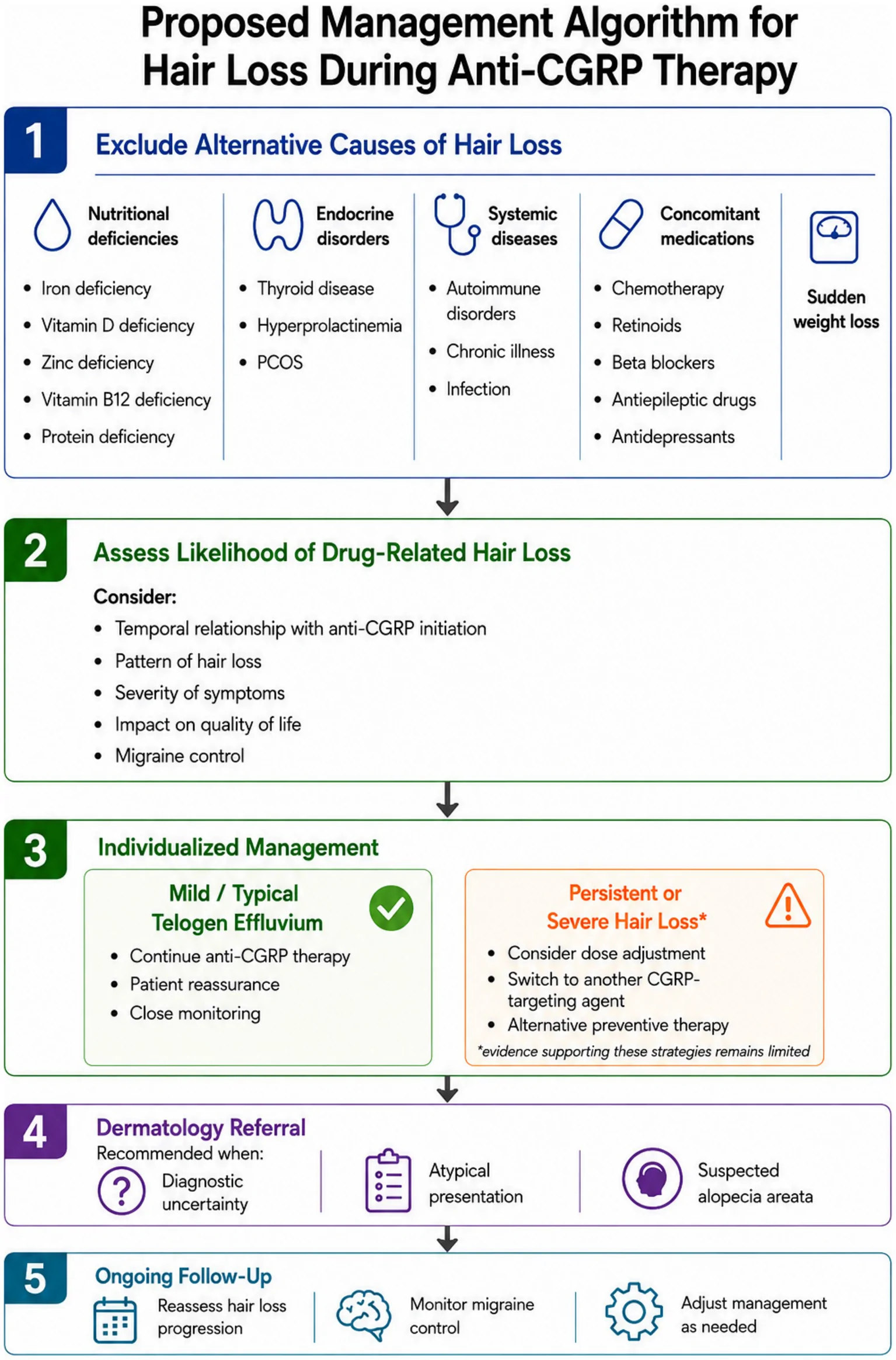
Proposed management algorithm for hair loss during anti-CGRP therapy.

Supportive treatment strategies generally follow standard approaches for non-scarring alopecia. Topical minoxidil remains the first-line therapy, while low-dose oral minoxidil has emerged as an off-label alternative with promising efficacy and tolerability (29–31). It promotes hair growth by prolonging the anagen phase, stimulating follicular activity and enhancing scalp microcirculation (32, 33). Drug efficacy is dependent on concentration, correct application, frequency of application and compliance. Men should apply 5% topical minoxidil solution or foam twice daily, whereas women are generally advised to apply the treatment once daily a couple of hours before bedtime to achieve optimal results (33, 34). Common adverse events associated with higher concentrations include pruritus, scalp irritation, allergic contact dermatitis, photoallergic reaction, hypertrichosis. Oral minoxidil should be avoided in patients with unstable angina, recent myocardial infarction or congestive heart failure (32, 33, 35). Minoxidil is contraindicated during pregnancy or breastfeeding. An initial hair shedding typically occurs within the first week of treatment initiation due to shortening of telogen phase and may persist for several weeks (32, 33, 36). Importantly, treatment requires long-term continuous use as clinical improvement typically becomes evident after 3–6 months, with maximal effects observed after 6–12 months of therapy (33, 37). Additionally, correction of underlying micronutrient deficiencies should be guided by laboratory findings, with particular attention to iron status and vitamin D levels (38, 39).

Importantly, patient counseling plays a central role. Patients should be informed that hair loss associated with one CGRP-targeting agent does not preclude the use of alternative CGRP inhibitors, as this adverse effect may not recur after switching therapies (14). This can help reduce anxiety and improve adherence to otherwise effective migraine therapy.

### 3.5 Strengths and limitations

This review has several strengths. It provides a focused, clinically oriented synthesis of the emerging evidence on hair loss associated with CGRP-targeting therapies, integrating case reports, case series, pharmacovigilance data and current insights into CGRP biology. Particular attention is given to the temporal relationship between treatment initiation and hair loss, recurrence after switching between agents, candidate biological mechanisms, alternative causes of alopecia and practical management considerations.

Several limitations should also be acknowledged. The available evidence is derived almost exclusively from case reports, case series and spontaneous pharmacovigilance reports (e.g., FAERS), which are inherently susceptible to reporting bias, incomplete clinical documentation and the absence of control groups. Disproportionality signals from such databases cannot establish causality or determine true incidence. A particular concern is the difficulty in distinguishing treatment-related alopecia from factors inherent to patients with chronic or severe episodic migraine. High disease burden may be accompanied by physiological and psychological stress, sleep disturbances, and polypharmacy, all of which may contribute to hair loss. As these factors are not adequately controlled for in spontaneous reporting systems such as FAERS, they may distort the observed association between CGRP-targeting therapies and alopecia. Finally, the mechanisms proposed to link CGRP inhibition to alopecia remain largely hypothetical, derived from experimental or extrapolated data rather than direct clinical evidence. These limitations warrant cautious interpretation of the current evidence and highlight the need for prospective studies with standardized dermatological assessment, systematic confounder evaluation and longer follow-up.

## 4 Conclusion

Alopecia has emerged as a potential safety signal associated with CGRP-targeting therapies, supported by a biologically plausible link to CGRP’s role in neuroimmune regulation, hair follicle immune privilege and cutaneous microcirculation. Reported cases are predominantly mild, non-scarring and reversible after drug discontinuation, and hair loss occurring with one agent does not appear to preclude switching to another within the class. Given the expanding use of these therapies, this potential adverse effect may carry meaningful implications, underscoring the need for clinical awareness, careful exclusion of alternative causes and individualized management decisions. As outlined above, current evidence remains constrained by its predominantly low-level, uncontrolled nature. Prospective studies with standardized dermatological assessment are needed to establish incidence, risk factors and the underlying causal mechanisms.
